# Panic attack triggering myocardial ischemia documented by myocardial perfusion imaging study. A case report

**DOI:** 10.1186/1755-7682-5-24

**Published:** 2012-09-21

**Authors:** Gastão Luiz Fonseca Soares-Filho, Claudio Tinoco Mesquita, Evandro Tinoco Mesquita, Oscar Arias-Carrión, Sergio Machado, Manuel Menéndez González, Alexandre Martins Valença, Antonio Egidio Nardi

**Affiliations:** 1 Panic and Respiration Laboratory, Institute of Psychiatry, Federal University of Rio de Janeiro. INCT - Translational Medicine (CNPq), Rio de Janeiro, Brazil; 2 Department of Nuclear Medicine, Pró-Cardíaco Hospital/PROCEP, Rio de Janeiro, Brazil; 3 Cardiology Department, Federal Fluminense University, Rio de Janeiro, Brazil; 4 Psychiatry Department, Medical School, Severino Sombra University, Vassouras, Brazil; 5 Movement Disorders and Transcraneal Magnetic Stimulation Unit, Hospital General Dr. Manuel Gea González, Secretaría de Salud, México; 6 Quiropraxia Program, Central University, Santiago, Chile; 7 Unit of Neurology, Hospital Álvarez-Buylla, Merida, Spain

## Abstract

**Background:**

Chest pain, a key element in the investigation of coronary artery disease is often regarded as a benign prognosis when present in panic attacks. However, panic disorder has been suggested as an independent risk factor for long-term prognosis of cardiovascular diseases and a trigger of acute myocardial infarction.

**Objective:**

Faced with the extreme importance in differentiate from ischemic to non-ischemic chest pain, we report a case of panic attack induced by inhalation of 35% carbon dioxide triggering myocardial ischemia, documented by myocardial perfusion imaging study.

**Discussion:**

Panic attack is undoubtedly a strong component of mental stress. Patients with coronary artery disease may present myocardial ischemia in mental stress response by two ways: an increase in coronary vasomotor tone or a sympathetic hyperactivity leading to a rise in myocardial oxygen consumption. Coronary artery spasm was presumed to be present in cases of cardiac ischemia linked to panic disorder. Possibly the carbon dioxide challenge test could trigger myocardial ischemia by the same mechanisms.

**Conclusion:**

The use of mental stress has been suggested as an alternative method for myocardial ischemia investigation. Based on translational medicine objectives the use of CO2 challenge followed by Sestamibi SPECT could be a useful method to allow improved application of research-based knowledge to the medical field, specifically at the interface of PD and cardiovascular disease.

## Background

Coronary artery disease (CAD) is one of the leading causes of death in the world [1] and demands prompt evaluation and proper treatment. The challenge is not only to diagnose acute coronary syndromes (ACS) in patients who present with the traditional combination of anginal chest pain and electrocardiography (ECG) changes but to recognize the presence of myocardial ischemia in situations of low probability.

Chest pain (CP) is a key element in the investigation of CAD. Although myocardial oxygen supply/demand imbalance may result in CP with no detectable atheromatous CAD, the vast majority of angina occurs in the presence of obstructive coronary artery plaques [2]. Panic disorder (PD), a strong component of mental stress (MS), is characterized by the occurrence of spontaneous and unexplained panic attacks (PA), which are periods of intense fear accompanied by somatic and cognitive symptoms, developed abruptly and reached a peak within 10 min.

Some somatic symptoms, so-called “respiratory symptoms” [3] of a PA are also seen in heart disease. These include choking/smothering sensations, shortness of breath, palpitation or accelerated heart rate, and CP. Along with palpitations, CP is associated with considerable disability and demand for medical resources [4]. Not surprisingly, patients presenting a PA very often seek emergency assistance. Usually, they are examined with a chest pain unit protocol [5] and released with the sole diagnosis that they are not presenting an ACS, without an investigation regarding possible psychiatric disorders. Although more than half of CP patients without ACS present an undiagnosed anxiety disorder [6], some studies have questioned the benign prognosis of PD.

The presence of PD is suggested as an independent risk factor for CAD, both as a trigger of myocardial infarction (MI) and the long-term prognosis of cardiovascular diseases [7]. Cases of PA leading to MI in patients with no evidence of CAD [8] have also been reported. Prospective epidemiological studies confirm a 3 to 6 fold increase risk of MI and of death [9,10]. Takotsubo cardiomyopathy [11], a syndrome that mimics an MI, has shown how acute stress can trigger myocardial damage without obstructive CAD. In a pioneer study, Fleet [12], using carbon dioxide (CO2) panic challenge, documented that PA could induce myocardial perfusion defects in patients who have CAD and PD, despite treatment with cardiac medications.

At this point, important questions arise: Is it really safe to inform a PD patient with chest pain that he’s not having a heart attack? Could PA induce myocardial perfusion defects in patients without known CAD? To introduce this discussion and faced with the extreme importance in differentiating from ischemic to non-ischemic chest pain, we report a case of PA induced by inhalation of 35% CO2 triggering myocardial ischemia, documented by myocardial perfusion imaging study.

## Methodology

The current study was approved by the Ethics Committee, consistent with the terms of the Helsinki Declaration. Written informed consent was obtained from the patient for publication of this Case report and any accompanying images. A copy of the written consent is available for review by the Editor-in-Chief of this journal. Informed consent was obtained from patient and his cardiologists.

In the course of our ongoing research, we examined a patient, without coronary risk factors and without cardiovascular medication, on their first visit to the Laboratory of Panic and Respiration, at the Institute of Psychiatry of the Federal University of Rio de Janeiro. He complained of chest pain and met criteria for PD after completing a structured interview based on the Diagnostic and Statistical Manual for Mental Disorders (DSM-IV), Fourth Edition [13]. Then, he was submitted to a technetium-99 m Sestamibi single-photon emission computed tomography (Sestamibi SPECT) investigation at rest and at maximum performance during a treadmill exercise test to rule out myocardial ischemia induced by physical stress. Once he showed no deficit in myocardial perfusion, he was invited to perform a Sestamibi SPECT following a carbon dioxide (CO2) panic challenge test.

It was proposed that a physiologic misinterpretation of a suffocation monitor can trigger an evolved suffocation alarm system [14]. This produces sudden respiratory distress followed promptly by brief hyperventilation and PA. CO2 is a rapid, potent stimulus to ventilation. Patients with PD are sensitive to small increases in CO2, presenting hyperventilation and PA, similar to the spontaneous presentation that occurs outside the laboratory setting [14].

Patient was submitted to a baseline Visual Analogue Scale for Anxiety (VAS-A) [15] to compare the level of anxiety before and after the CO2 challenge. To record hemodynamic data, he was outfitted with a 12-lead electrocardiograph, a sphygmomanometer, and a pulse oximeter. A catheter was inserted for injection of the radioisotope. Patient rested for 10 min in a quiet room, while baseline heart rate (HR), blood pressure (BP), and oxygen saturation (OS) were recorded. After CO2 challenge, vital signs were sequentially recorded every 20 seconds for four minutes. The double product (HR × systolic BP) was used to estimate myocardial work and oxygen consumption.

The CO2 panic challenge consisted of two sequential vital capacity inhalations of a gas containing 35% CO2 and 65% oxygen (O2), delivered through a facial mask. After the patients completed the VAS-A post-CO2 challenge, the Panic Symptom Scale (PSS) was administered [16]. This survey asks subjects to rate the intensity (absent, mild, moderate, severe, or extremely severe) of 13 panic symptoms derived from DSM-IV. Patients were classified as having had a PA if they reported have experienced 4 or more of the 13 panic symptoms of PSS.

Immediately after second gas inhalation, Technetium-99 m Sestamibi was injected as a marker of myocardial perfusion, regardless of whether or not patients presented a PA. SPECT acquisition was performed and independently interpreted by nuclear cardiology specialists.

## Case report

JCN, a 55-year-old man, sought assistance due to a long life history of agoraphobia related to bridges, tunnels, and traffic. He rarely left his house alone. In the previous weeks, he had experienced spontaneous panic attacks even at home. These attacks were characterized by chest pain, dizziness, sensation of suffocation, and paresthesia.

The day he came to the panic experiment, the patient scored 5 points on the VAS-A before the test. After the test, the patient scored 6 points. At PSS, he reported mild chest pain, palpitations and dizziness, but he denied that he had experienced a panic attack.

In the pre-challenge phase, his HR was 78 bpm. At twenty seconds, it increased to 89 bpm, decreasing to 83 bpm at minute one. His baseline BP was 140×80 mm/Hg. This value has changed in 20 seconds, increasing to 160×90, up to 190×100 in a minute, when his double product reached the peak value of 15.770 bpm × mm/Hg. The oxygen partial pressure (PO2) did not change after the test (Table 1).

**Table 1:** Stress and hemodynamic measurement

**Measures**	**Before**	**20 s after**	**1 min after**	**4 min after**
**VAS-A**	5	6	6	6
**PSS**		Mild chest pain, palpitations and dizziness	Denied panic attack	
**HR (BPM)**	78	89	83	74
**BP (mm/Hg)**	140/80	160/90	190/100	160/80
**PO2 (%)**	98	96	98	90
**DP (bpm x mm/Hg)**	10,920	14,240	15,770	11,840

**Abbreviations:** VAS-A= Visual Analogue Scale for Anxiety; PSS=Panic Symptoms Scale; HR= Heart Rate; BP= Blood Pressure; PO2= Pulse Oximetry; DP= Double Product.

**Figure 1:**
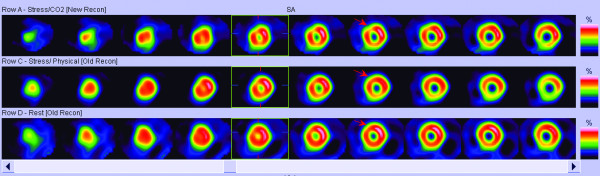
SPECT images of myocardial perfusion.

The ECG did not change after the test, but SPECT images analyzed by two nuclear cardiologists demonstrated a reversible myocardial perfusion defect in mid antero-septal segment consistent with myocardial ischemia (Figure 1).

## Discussion

Panic attacks can cause CP by way of several cardiac mechanisms [17], often involving the presence of hyperventilation. The increase in respiratory rate (RR), inducing an alkalotic state, can trigger intracellular influx of calcium ions and provoke coronary vasospasm and myocardial ischemia [18]. Positive hyperventilation test patients are likely to have life-threatening arrhythmias and multivessel spasms during attacks [19].

Panic disorder patients with prominent respiratory symptoms can be more sensitive to CO2 challenge and consequentially have more severe panic and phobic symptoms [20]. The patient described in this case report conveyed very few symptoms and denied having a PA as he had experienced before, but did present myocardial perfusion deficit. Analyzing hemodynamic and psychometric data recorded during test, important questions in the field of mental stress and myocardial ischemia are raised.

Patients with CAD may present myocardial ischemia as a consequence of two possible mechanisms present in the MS response: an increase in coronary vasomotor tone with decreased coronary blood flow or a sympathetic hyperactivity that determines an increase in HR, BP and myocardial contractility, leading to a rise in myocardial oxygen consumption [21-23].

Ischemia induced by MS is often silent [24], as happened in the case reported and usually occurs with HR and consequently double product (DP) lower than that found in exercise-induced ischemia [25]. The patient experienced a DP during treadmill exercise-test peak effort greater than that presented in the CO2 challenge test (32,550 ×15,770), with no evidence of ischemia in ECG or perfusion deficit on Sestamibi-SPECT. This evidence suggests the presence of a primary reduction of coronary flow, associated with a rise in myocardial oxygen consumption.

Recently, endothelial dysfunction has been described as a potential contributor to the development of coronary artery spasm and reduction of coronary flow, by reducing the secretion of relaxation factor by the endothelium [26]. In healthy individuals, with intact endothelium, MS normally induces coronary artery vasodilation as a response to sympathetic stimulus [27]. This vasodilation in normal arteries appears to be mediated by the stimulation of α2 adrenergic receptors, which promote the release of nitric oxide. In CAD individuals, endothelial injury blocks these events, enabling MS to trigger coronary vasoconstriction and myocardial oxygen offer/demand imbalance [28,29]. Coronary artery spasm was presumed to be the mechanism involved in three cases of cardiac ischemia linked to PD [30]. Indeed, angiography has documented coronary vasoconstriction at the site of atherosclerosis during the arithmetic performance test [31].

Myocardial perfusion deficits after MS were documented in subjects with normal exercise or chemical nuclear stress test results [32]. Possibly CO2 challenge test could trigger myocardial ischemia by the same mechanisms. About 30% of CP patients arriving at the emergency room have PD [33]. Among this group, 22.4% exhibited PD with no CAD identified with traditional investigation. Are they really free of cardiovascular risk?

Notably, the patient described in this case report denied having a PA but experienced changes in myocardial scintigraphy. The patient complained of mild chest pain, palpitations and dizziness. Hemodynamic data otherwise showed rises in systolic BP and HR, suggesting adrenergic hyper-activity. Beitman et al. [34], studying chest pain patients without CAD, found that between 32% and 41% had PD without experiencing the feeling of fear. Fleet et al. in his aforementioned work [12] found that around 50% of controls, who did not develop a PA, displayed perfusion defects with the CO2 challenge. He has proposed three possible explanations: a startle response inducing perfusion defect, a redistribution of blood flow secondary to a CO2-induced vasodilatation in certain coronary vessels and a tendency for patients in the control group to deny panic symptoms.

The use of MS has been suggested as an alternative method for myocardial ischemia investigation [35]. There is no doubt about the ability of a PA to act as a powerful mental stressor. Based on translational medicine objectives [36] of an integrated application of innovative tools, clinical methods, and technologies to improve the understanding of medical disorders, the use of CO2 challenge followed by Sestamibi SPECT could be a useful method to allow improved application of research-based knowledge to the medical field, specifically at the interface of PD and cardiovascular disease.

## Conclusion

To our knowledge, this is the first time myocardial perfusion has been studied in laboratory panic attacks in a population free of known CAD. More studies are currently being performed to investigate radionuclide imaging applicability in PD patients with prominent cardiovascular symptoms.

## Competing interests

The authors declare that they have no competing interests.

## Authors’ contributions

GLFSF, CTM, ETM, AMV and AEN designed, conducted the literature review and drafted most of the manuscript. GLFSF, CTM, ETM, OAC, SM, AMV and AEN performed the literature review and the drafting of the manuscript. All authors were equally involved in reading and approving the final manuscript.
